# Exposure to Exogenous Enkephalins Disrupts Reproductive Development in the Eastern Lubber Grasshopper, *Romalea microptera* (Insecta: Orthoptera)

**DOI:** 10.1371/journal.pone.0051126

**Published:** 2012-11-30

**Authors:** Sandeep Kumar, Purnachandra Nagaraju Ganji, Hojun Song, Laurence von Kalm, David W. Borst

**Affiliations:** 1 Department of Biology, University of Central Florida, Orlando, Florida, United States of America; 2 Department of Hematology and Medical Oncology, Winship Cancer Institute, Emory University, Atlanta, Georgia, United States of America; 3 Biomolecular Sciences Center, University of Central Florida, Orlando, Florida, United States of America; Instituto Butantan, Brazil

## Abstract

Enkephalins play a major role in reproductive physiology in crustaceans; however their role in reproductive development in insects is largely unknown. We investigated the effect of exposure to exogenous leucine-enkephalin (Leu-Enk), methionine-enkephalin (Met-Enk), and the opioid antagonist naloxone on gonad development in the Eastern lubber grasshopper, *Romalea microptera*. Injection of either Leu-Enk or naloxone alone significantly increased the testicular index and testicular follicular diameter in males, and the ovarian index, oocyte length, and oocyte diameter in females. In contrast, injection of Met-Enk inhibited all measures of reproductive development in both sexes. Surprisingly, co-injection of naloxone with either enkephalin enhanced the effect associated with administration of the enkephalin alone. This study clearly demonstrates the ability of enkephalins to disrupt insect sexual development and also suggests the existence of conserved enkephaline-dependent regulatory mechanisms in insects and crustaceans.

## Introduction

Enkephalins, classified under endorphins, are opioid penta-peptides involved in nociception through opiate receptors [1]. The two major types of enkephalins, leucine-enkephalin (Leu-Enk, Tyr-Gly-Gly-Phe-Leu) [2] and methionine-enkephalin (Met-Enk, Tyr-Gly-Gly-Phe-Met) [3], are the products of the proenkephalin gene [4], [5], widely present in the animal kingdom from bivalve molluscs (e.g. *Mytilus edulis*) and annelids (e.g. *Theromyzon tessulatum*) to amphibians (frog) and mammals (mice) [6]. Enkephalins have been widely studied in different groups of vertebrates and invertebrates [7] and are expressed in the central nervous system (CNS) of many animals including humans where they act as neurotransmitters or neuromodulators [8]. Apart from nociception, opiates are also involved in inducing euphoria, cardiovascular regulation, decrease in gastrointestinal motility, susceptibility to seizures, and food consumption behavior [9].

Among invertebrates, the role of exogenous enkephalins in reproductive biology has been studied in decapod crustaceans in depth [10]. In general, Leu-Enk and Met-Enk have opposite effects on reproductive indices (Reproductive/Gonad Indices (GI) = (weight of gonads/weight of animal) X 100) in crustaceans with Leu-Enk acting positively and Met-Enk acting negatively. For example, in the prawn *Penaeus indicus,* Reddy *et al*. [11] reported an increase in reproductive indices following Leu-Enk injection, and a decrease following Met-Enk injection. Similarly, Kishori *et al*. [12] reported increased ovarian growth and vitellogenesis in the rice field crab *Oziotelphusa senex senex* following Leu-Enk administration, and Sarojini *et al.* [13] reported a dose dependent delay in ovarian maturation in the female crab *Uca pugilator* after exogenous Met-Enk administration.

In insects, indirect evidence for enkephalin-like neuropeptides has been reported [14], [15], but there has not been a study on the effect of exogenous enkephalins. Immunoreactive enkephalin-like neuropeptides have been observed in the nervous system of the locust *Locusta migratoria* [10] and blowfly *Calliphora vomitoria* [16], and in the corpus cardiacum and corpus allatum of the cockroach *Leucophaea maderae* [17]. Met-Enk immunoreactivity has also been reported in the gonads of *L. migratoria* and *Sarcophaga bullata* [10], suggesting a possible role for opioid peptides in insect reproductive physiology. However, there is no report demonstrating a clear influence of enkephalins on insect reproductive development. Furthermore, endogenous enkephalins have not been purified from either insects or crustaceans.

Exposure to exogenous naloxone, an opioid antagonist [19], has been shown to elevate luteinizing hormone (LH) and testosterone levels in rats [20]. It has also been reported to enhance ovarian index in crustaceans [21]. For example, Cahansky *et al*. [22] reported an increase in lipid concentration associated with reproductive growth in the ovaries of the cockroach crayfish *Aegla platanis* after ingestion of naloxone containing food. Recently, naloxone has been found to bind with retinoid X receptors (RXR) [23], which are associated with reproduction [24], suggesting that naloxone may also play a role in reproductive physiology in crustaceans and vertebrates. However, the effect of naloxone on reproduction and its interaction with enkephalins has not been investigated in insects. In this study we hypothesized that the effects of exposure to exogenous enkephalins in insects would be similar to previously reported studies in crustaceans. While the hypothesis is supported for Leu-Enk and Met-Enk, we find unexpectedly that naloxone enhances the effect of both enkephalins in the Eastern lubber grasshopper *Romalea microptera* (Insecta: Orthoptera), which has been used as a model organism for insect physiology and reproductive endocrinology [25], [26], [27], [28].

**Figure 1:**
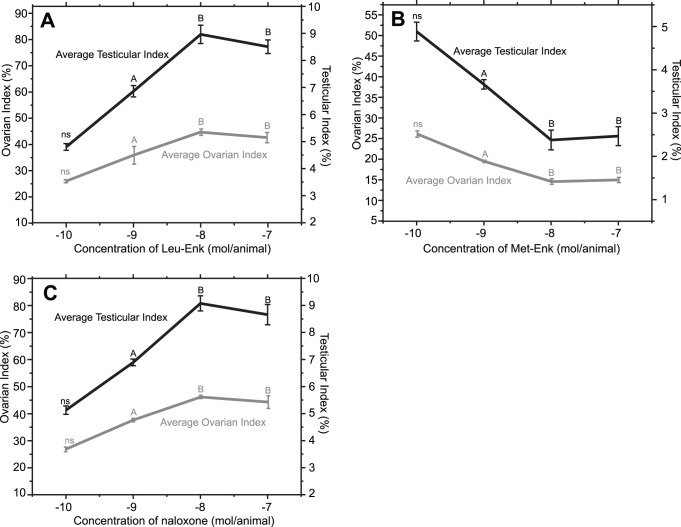
Dose dependent effects of Leu-Enk (A), Met-Enk (B), and naloxone (C) on average ovarian index in female and average testicular index in male grasshoppers. The left y-axis indicates ovarian index while the right y-axis indicates testicular index. ‘ns’ indicates statistical non-significance (*p*>0.05) compared to the control (not shown). The measurements for the control was 26.15±0.46% for ovarian index and 5.05±0.2% for testicular index. The letters indicate statistical significance from the control (*p*<0.05). ‘A’ and ‘B’ are also statistically different from each other at *p*<0.05.

## Materials and Methods

Grasshoppers were collected from in and around University of Central Florida, Orlando, Florida (No specific permits were required for the collection of grasshoppers and studies) and were reared for two generations in laboratory conditions at 28±2°C in a 16:8 L:D cycle. Food consisted of Romaine lettuce and wheat bran offered *ad libitum* until the final molt to adult instar. Leu-Enk, Met-Enk, and naloxone were purchased from Sigma (Sigma Aldrich, St. Louis, MO, USA) in a powder form and were dissolved in 1X PBS prior to use.

A total of one hundred and forty adult grasshoppers (third generation, laboratory reared) were used and divided into 14 groups of 10 insects each (N = 10). Two groups were used as control (no treatment) and another two groups were used as concurrent control (injection with 1X PBS). The remaining 10 groups were further divided into five treatment groups (each with two groups or 20 insects): Leu-Enk only, Met-Enk only, naloxone only, Leu-Enk + naloxone, and Met-Enk + naloxone. For each treatment, grasshoppers were injected on the first, fifth, tenth, and fifteenth day from adult emergence at a fixed dose of 10^−8 ^mol/grasshopper in 10 µL volume per injection. No significant mortality was observed in either the control or experimental groups, and the grasshoppers were sacrificed on the 20^th^ day from adult emergence. Animals were first immobilized with carbon dioxide to measure body weight. Then, reproductive organs were dissected into 1X PBS and excessive fat body was removed. Organs were lightly blotted with paper towel and weighed on an electronic balance. Gonad indices (GI) for oocytes and testicular follicles were measured using the following formula: GI = (weight of gonads/weight of animal) X 100. The linear dimensions of the reproductive structures were measured using an ocular micrometer attached to a Leica MZ6 microscope.

For dose-dependent studies of Leu-Enk, Met-Enk and naloxone, male and female insects were injected with 10^−10^mol/grasshopper, 10^−9^mol/grasshopper, 10^−8^mol/grasshopper, and 10^−7^mol/grasshopper in 10 µl volume. Gonadal indices were measured for each dose as described above.

For statistical analyses, one-way ANOVA followed by Tukey-Kramer multiple comparison test was used to compare different treatments.

**Figure 2:**
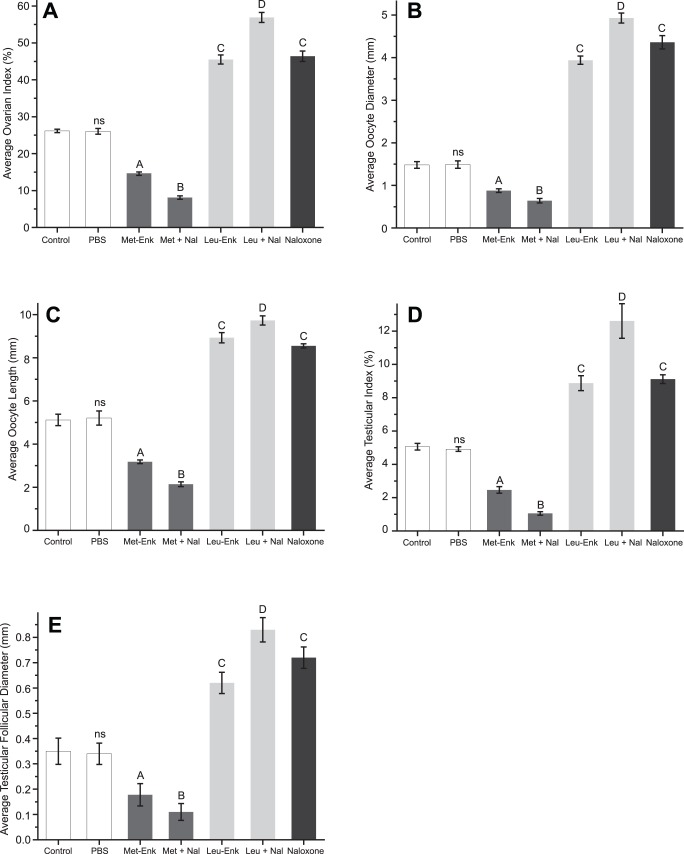
Effect of enkephalins alone, naloxone alone or combinations on ovarian index (A), average oocyte diameter (B), average oocyte length (C) of female grasshoppers, and average testicular index (D) and average testicular follicular diameter (E) of male grasshoppers. ‘ns’ indicates statistical non-significance (*p*>0.05) compared to the control and the letters indicate that the effects of various doses were significantly (*p*<0.05) different from each other and the control.

## Results

To investigate the role of enkephalins and naloxone in *Romalea* reproductive physiology we first studied the dose-dependent effects of Leu-Enk, Met-Enk, and naloxone when administered singly (Figure 1). For all three compounds, an optimal dose of 10^−8^ mol/animal was observed.

For Leu-Enk, ovarian index increased in a dose-dependent manner up to a concentration of 10^−8^ mol/animal (Figure 1A). A higher dose (10^−7^ mol/animal) did not yield further development. Similar results were observed for testicular index (Figure 1A) as well as average oocyte diameter, average oocyte length and average testicular follicular diameter (data not shown). As previously documented in crustaceans [11], the opposite result was observed following administration of Met-Enk where ovarian index and testicular index both declined with increasing dose (Figure 1B). Naloxone, an opioid antagonist, had a similar dose-dependent profile to Leu-Enk with an increase in all reproductive indices observed as dose increased (Figure 1C). Similarly to Leu-Enk, the concentration of 10^−8^ mol/animal appeared to be the maximally sensitive dose for Met-Enk and naloxone.

To investigate potential interactions between naloxone and Leu-Enk or Met-Enk, we performed experiments with animals injected with two compounds. A similar profile was observed for all reproductive indices measured, where a combination of enkephalin with naloxone served to enhance the effect of administration of the enkephalin alone (Figure 2). For example, a combination of Met-Enk and naloxone significantly reduced the ovarian index when compared to Met-Enk alone (Figure 2A). Thus, naloxone, which has a positive effect on reproductive indices when administered alone, acts negatively in the presence of Met-Enk. In contrast, a combination of Leu-Enk and naloxone significantly increased ovarian index when compared to either Leu-Enk or naloxone alone (Figure 2A).

## Discussion

In this study we demonstrate that exposure to exogenous enkephalins influences reproductive development in *Romalea microptera*. As previously reported in decapod crustaceans [29], we find an antagonistic interaction between Leu-Enk and Met-Enk in terms of reproductive development. Leu-Enk stimulated gonad development in both male and female grasshoppers, while Met-Enk had the opposite effect. The biological effects of both enkephalins were dose-dependent with a maximum effect observed at 10^−8^ mol/animal. Our findings are consistent with the observations of Schoofs *et al.* [10] who reported immunoreactivity to Met-Enk in the gonads of two distantly related insect species, *L. migratoria* and *S. bullata*, and speculated that Met-Enk might play a role in insect reproductive physiology. In contrast, while Schoof *et al.* [10] observed immunoreactivity against Leu-Enk in the central nervous system of both species, immunoreactivity was not detected in the ovaries. This raises the possibility that enkephalins or enkephalin-like peptides may regulate insect reproduction indirectly via the neuroendocrine system. In support of this hypothesis, Duve and Thorpe [16] reported the presence of enkephalin-like peptides in the brain, corpus cardiacum, and corpus allatum in the blowfly *Calliphora vomitoria*.

Naloxone has been previously reported to enhance reproductive indices in crustaceans [21], [22], [30] and our work extends this finding to insects. Because naloxone is an opioid antagonist, we expected that it would negate the effect of both enkephalins when co-administered. Instead, naxolone acted as a synergistic agonist of both enkephalins in our study, significantly increasing the stimulating effect of Leu-Enk and enhancing the inhibitory effect of Met-Enk. These data are interpretable in a context where naloxone increases receptor sensitivity to enkephalins. Schulz *et al.* [31] reported that chronic injection of naloxone in guinea pigs resulted in an increased sensitivity to Met-Enk action in muscle mesenteric plexus of ileum. Similarly, Tang and Collins [32] reported the enhancement of analgesic action of morphine following chronic administration of naloxone in rats. The mode of action for naloxone in *R. microptera* is unknown; however naloxone has been reported to stimulate the action of gonad stimulating hormone (GSH) in the red claw crayfish [22]. Further studies are needed to determine the mode of action of naloxone and the mechanism of its interaction with enkephalins in insects.

To date there is no direct evidence for the existence of Leu- and Met- enkephalins in insects or crustaceans. Indirect evidence is based on cross-immunoreactivity with mammalian antibodies [7], [16], [18], or exogenous application of these compounds [11], [12], [13]. The effect of exogenous enkephalins on reproductive development has been studied in depth in crustaceans as reviewed by Nagaraju [29], and our findings are consistent with the previous reports in crustaceans. The similarity between our observations and those reported in crustaceans imply the possible existence of a conserved enkephalin response mechanism in arthropods. This suggests that the response to enkephalins may be an ancestral trait that evolved before the divergence of hexapods and decapods. In our opinion it seems unlikely that an artifactual response to exogenous administration of enkephalins would be conserved throughout the Pancrustacea unless the enkephalins were interacting with molecules in another highly conserved neuroendocrine pathway. Clearly, further studies now require a definitive demonstration of the existence of endogenous enkephalins in insects and crustaceans. If endogenous enkephalins are identified, it will be interesting to determine how they interact with juvenile hormone, ecdysone, ecdysteroids, and gonadotropic neurohormones such as allatotropin and allatostatin to regulate insect reproductive physiology.
