# Correction: Exposure to Exogenous Enkephalins Disrupts Reproductive Development in the Eastern Lubber Grasshopper, *Romalea microptera* (Insecta: Orthoptera)

**DOI:** 10.1371/annotation/7c19edf3-4648-45de-8132-16092fc4bf40

**Published:** 2013-05-01

**Authors:** Sandeep Kumar, Purnachandra Nagaraju Ganji, Hojun Song, Laurence von Kalm, David W. Borst

There was an error in the citation. The correct citation is:

Kumar S, Nagaraju GP, Song H, von Kalm L, Borst DW (2012) Exposure to Exogenous Enkephalins Disrupts Reproductive Development in the Eastern Lubber Grasshopper, Romalea microptera (Insecta: Orthoptera). PLoS ONE 7(11): e51126. doi:10.1371/journal.pone.0051126 

